# Comprehensive Genome-Wide Identification and Transcript Profiling of GABA Pathway Gene Family in Apple (*Malus domestica*)

**DOI:** 10.3390/genes12121973

**Published:** 2021-12-12

**Authors:** Qingbo Zheng, Shenghui Su, Zhe Wang, Yongzhang Wang, Xiaozhao Xu

**Affiliations:** 1 College of Horticulture, Qingdao Agricultural University, Qingdao 266109, China; qingbozheng@ibcas.ac.cn (Q.Z.); ssh8601@163.com (S.S.); 2 Engineering Laboratory of Genetic Improvement of Horticultural Crops of Shandong Province, Qingdao Agricultural University, Qingdao 266109, China; wangzhe19970502@163.com; 3 Laboratory of Quality & Safety Risk Assessment for Fruit (Qingdao), Ministry of Agriculture and Rural Affairs, Qingdao Agricultural University, Qingdao 266109, China; wangyzh304@163.com

**Keywords:** apple, GABA, transcript

## Abstract

γ-Aminobutyric Acid (GABA), a four-carbon non-protein amino acid, is a significant component of the free amino acid pool in most prokaryotic and eukaryotic organisms. GABA is involved in pH regulation, maintaining C/N balance, plant development and defence, as well as a compatible osmolyte and an alternative pathway for glutamate utilization via anion flux. Glutamate decarboxylase (GAD, EC 4.1.1.15) and GABA transaminase (GABA-T, EC 2.6.1.19) are two key enzymes involved in the synthesis and metabolism of GABA. Recently, GABA transporters (GATs), protein and aluminium-activated malate transporter (ALMT) proteins which function as GABA receptors, have been shown to be involved in GABA regulation. However, there is no report on the characterization of apple GABA pathway genes. In this study, we performed a genome-wide analysis and expression profiling of the GABA pathway gene family in the apple genome. A total of 24 genes were identified including five GAD genes (namely *MdGAD 1–5*), two GABA-T genes (namely *MdGABA-T 1*,*2*), 10 GAT genes (namely *GAT 1–10*) and seven ALMT genes (namely *MdALMT* *1–7*). These genes were randomly distributed on 12 chromosomes. Phylogenetic analyses grouped GABA shunt genes into three clusters—cluster I, cluster II, and cluster III—which had three, four, and five genes, respectively. The expression profile analysis revealed significant *MdGAD4* expression levels in both fruit and flower organs, except pollen. However, there were no significant differences in the expression of other GABA shunt genes in different tissues. This work provides the first characterization of the GABA shunt gene family in apple and suggests their importance in apple response to abiotic stress. These results can serve as a guide for future studies on the understanding and functional characterization of these gene families.

## 1. Introduction

γ-Aminobutyric Acid (GABA) is a four-carbon amino acid; however, it is not an α amino acid and does not incorporate into proteins. GABA includes an amino group on the γ-carbon and is mostly found as a zwitterion, with both positive and negative electrical charges [1]. GABA is a flexible molecule substantial for many biological and physiological events such as inter and intracellular transformation, interaction with other biological signals [2,3], as well as the regulation of cell wall modification and gene expression [4,5,6].

In plants, numerous studies have revealed that GABA rapidly accumulates in response to biological and abiotic stresses [7], including temperature [8] (Zhu et al., 2019), osmotic pressure [6,9], and high salinity [10,11,12]. For example, the overexpression of GAD gene in *Nicotiana sylvestris*, application of exogenous GABA to muskmelon (*Cucumis melo*) and lettuce (*Lactuca sativa*) seedlings and GABA-T deficient in *Arabidopsis* have been shown to contribute to salt stress tolerance [6,11,12,13,14]. In addition, GABA has also been shown to regulate plant growth and development [3,15]. In tomato, the removal of the C-terminal autoinhibition region (calmodulin-binding domain) of *SlGAD* increases GABA levels and wild type levels in fruits, and causes slow growth, more branched and shorter cortical parenchyma cell elongation and promoted fruit ripening [16]. Cell elongation was severely impaired in *Arabidopsis* pollen tubes, primary root, and hypocotyls when the GABA transaminase (GABA-T) gene was disrupted, leading to elevated tissue GABA concentrations [5,11]. In poplar, endogenous GABA accumulation interferes with hormone transport and carbon/nitrogen metabolism, which delays root primordia formation and inhibits root elongation [17,18].

The synthesis and metabolic pathway of GABA have been referred to as GABA shunt associated with TCA, which is a feedback loop producing and conserving GABA supply [11,19,20]. In general, two pathways are involved in GABA synthesis and metabolism, where the main pathway starts with the decarboxylation of Glu to produce GABA and CO_2_ by the GAD enzyme in the cytosol [20]. The second pathway is catalyzed by GABA-T in a reversible transamination, which produces succinic semialdehyde (SSA). In addition, GABA, as a signal molecular, plays a variety of functions by transport and receptors of GABA. Aluminium-activated malate transporter (ALMT) proteins were first reported as a GABA receptor and activated by anions and negatively regulated by GABA in plant [15]. GABA exerts its multiple physiological effects in plants via ALMT, including the regulation of pollen tube and root growth, therefore GABA can finally be considered a legitimate signalling molecule in both the plant and animal kingdoms [3,15,21].

GABA transporters (GATs), which belong to six of the amino acid/auxin permease (AAAP) subfamilies [22], mainly transport the ubiquitous amino acid across cellular membranes in flowering plants and play indispensable roles in various processes of plant growth and development [23]. *LeProT1* is an efficient transporter for glycine betaine and the stress-induced GABA in tomato [24]. *AtGAT1*, the first high affinity GABA transporter protein, was reported in *Arabidopsis*. Characterization in heterologous expression systems showed that the kinetic properties and substrate selectivity of *AtGAT1* are similar but distinct from mammalian, bacterial, and *Saccharomyces cerevisiae* GABA transporters [25]. Consistent with a role in GABA uptake into cells, transient expression of *AtGAT1*/green fluorescent protein fusion proteins in tobacco protoplasts revealed localization at the plasma membrane [25]. *PeuGAT3* increased the thickness of xylem cells walls in both Arabidopsis and poplar and enhanced the lignin content of xylem tissues and the proline accumulation in poplar leaves, all of which may improve tolerance of salt/drought stress in desert poplars [26]. In addition, more putative GAT-like proteins were identified in the genome of *Populus* species [26,27,28].

The domesticated apple (*Malus × domestica* Borkh) is one of the most important fruit crops, widely distributed in temperate regions of the world. Recently, the genome of the domesticated apple was fully sequenced based on the doubled haploid GDDH13 [29,30]. This information provides an opportunity to further analyze the GABA pathway gene family in apple. 

In this study, we identified 24 GABA pathway genes in the apple genome and investigated their phylogenetic relationships. Furthermore, the expression pattern and subcellular localization of the 24 genes were also investigated. Our results reveal the molecular characteristics and evolutionary pattern of the GABA pathway gene family and provide a foundation for future elucidation of the biological functions of GABA pathway genes in apples.

## 2. Materials and Methods

### 2.1. Identification and Classification of the Apple GABA Pathway Genes 

We downloaded the apple genome data (GDDH13) from the Genome Database for Rosaceae (GDR, https://www.rosaceae.org/, 10 September 2020). Protein sequences of the Arabidopsis GABA pathway proteins were obtained from The Arabidopsis Information Resource (TAIR, https://www.arabidopsis.org/, 9 October 2020). Apple GABA pathway genes were searched using the two basic local alignment search tool (BLAST) methods to identify the maximum number of GABA pathway genes. Then, primary GABA pathway proteins were identified using blast methods in UniProKB/Swiss-Prot database and the conserved domains were searched using CD-search on the NCBI website (https://www.ncbi.nlm.nih.gov/Structure/cdd/wrpsb.cgi, 11 October 2020).

### 2.2. Phylogenetic Analysis of the GABA Pathway Proteins 

The phylogenetic tree was constructed using Arabidopsis and apple GABA pathway full length protein sequences in MEGA7.0 (Arizona State University, Tempe, AZ, USA) with the Maximum Likelihood (ML) method with the following parameters: 1000 bootstrap test replicates, Jones-Taylor-Thornton (JTT) model, Uniform rates, Complete deletion, and Nearest-Neighbor-Interchange (NNI). 

### 2.3. Chromosomal Distribution and Synteny Analysis

All GABA pathway genes were mapped to apple chromosomes using Amazing Gene Location From GTF/GFF by TBtools (https://github.com/CJ-Chen/TBtools, 15 October 2020) [31]. Synteny blocks between Apple and Arabidopsis genomes as well as within the apple genome were determined by Quick MCScanX Wrapper and visualized by Dual Synteny Plotter in TBtools. 

### 2.4. Sequences Analysis

Conserved motifs in GABA pathway proteins were analyzed using the MEME (http://meme-suite.org/, 19 October 2020) web server [32] (Bailey et al., 2009). The structures of GABA pathway genes were determined using Amazing Optional Gene Viewer in TBtools. The 2000 bp sequences of the GABA pathway genes start site (ATG) from the Genome Database for Rosaceae (https://www.rosaceae.org/, 25 October 2020) were downloaded and the cis-elements of promoters were identified by PlantCARE [33].

To predict the subcellular localization of GABA pathway proteins, simply paste protein’s amino acid sequence (single letter code) in the WoLF PSORT window below and click submit (https://www.genscript.com/wolf-psort.html?src=leftbar, 30 October 2020).

### 2.5. GABA Pathway Genes Expression Profiles

The expression profiles of GABA pathway genes were determined in *Malus domestica* gene expression atlas of various organs. RNA-seq data sets (SRA accession no. SRP125281, SRP102870, and SRP050139) were retrieved from NCBI SRA database (https://www.ncbi.nlm.nih.gov/sra, 8 November 2020). The expression patterns of apple GABA pathway genes in fruit developmental stages and bud development were acquired from NCBI SRA database (SRA accession no. SRP018878, SRP034165, SRP099578). To investigate the expression profiles of apple GABA pathway genes in response to different stress treatment (*Penicillium expansum*, *Venturia inaequalis*, Apple Stem Grooving Virus, Iron deficiencies, and Phosphorus deficiencies), apple RNA-seq data sets (SRA accession no. SRP150975, SRP034943, and SRP018878) were retrieved from NCBI SRA database (https://www.ncbi.nlm.nih.gov/sra, 12 November 2020).

The analysis of RNA-seq data was according to previous method and the FPKM (Fragments Per Kilobase of exon model per Million mapped fragments) were used to estimate the gene expression level. The heatmap of apple GABA pathway genes was exhibited using TBtools [34].

## 3. Results

### 3.1. Genome-Wide Identification and Characterization of GABA Pathway Gene Family Members in Apple 

In the apple genome, a total of 24 putative genes involving GABA biosynthesis, catabolism, transports and receptors were identified. They were designed as *MdGAD*, *MdGABA-T*, *MdGAT* and *MdALMT*, respectively, according to different physiological and biochemical properties (Table 1). In addition, 24 putative genes sequence data can be found in Table S1. Detailly, five putative glutamate decarboxylases namely MdGAD that catalyzed glutamate conversion to produce GABA were identified. The length of the MdGAD proteins ranged from 333 to 510 amino acids, with predicted molecular weights of 37.68 kDa to 56.95 kDa. The theoretical isoelectric point (pI) of the MdGAD proteins ranged from 4.95 to 5.71, and MdGAD1-4 protein subcellular localization predicted in the cytoplasmic, and MdGAD5 was predicted in the chloroplast; two putative GABA transaminase namely *MdGABA-T* genes that catalyzed GABA conversion to produce succinic semialdehyde (SSA) were identified. The length of the two MdGABA-T proteins was 514 and 521 amino acids, with predicted molecular weights of 56.69 kDa and 57.59 kDa. The theoretical isoelectric point (pI) of the two MdGABA-T proteins was same 6.95, and MdGABA-T protein subcellular localization predicted in the chloroplast; 10 putative GABA transporters namely *MdGAT* genes that transport GABA from cell to cell were identified. The length of the MdGAT proteins ranged from 174 to 510 amino acids, with predicted molecular weights of 19.42 kDa to 56.40 kDa. The theoretical isoelectric point (pI) of the MdGAT proteins ranged from 4.79 to 9.88, and MdGAT protein subcellular localization predicted in the cytoplasmic, chloroplast, vacuole, and inner membrane; Aluminum-activated malate transporters (ALMTs) play an important role in aluminum tolerance, stomatal opening, and fruit acidity in plants. A total of 25 *MdALMT* genes were identified from the apple reference genome of the “Golden Delicious” doubled-haploid tree (GDDH13) (Figure S1a) [35]. According to previous studies, ALMT proteins, which is the presence of a putative GABA-binding motif, are key transducers of GABA signaling in plants [15,36,37], Based on a putative GABA-binding motif, 12 amino acids in length shared between ALMTs participated in GABA signaling in plants [15]. Seven putative GABA receptors genes, namely *MdALMT1*, *MdALMT2*, *MdALMT4*, *MdALMT5*, *MdALMT14*, *MdALMT17*, and *MdALMT20* that may play core roles in GABA signal transduction were identified in the apple genome (Table 1), which is closely related to TaALMT1 through the phylogenetic tree (Figure S1b) and contained the putative conserved GABA-binding motif (Figure S1c). The length of the seven putative GABA receptors MdALMT proteins ranged from 472 to 493 amino acids, with predicted molecular weights of 51.81 kDa to 53.85 kDa. The theoretical isoelectric point (pI) of the seven MdALMT proteins ranged from 8.21 to 9.39, and all the seven MdALMT protein subcellular localizations were predicted in the inner membrane (Table 1).

### 3.2. Chromosome Distribution and Duplication of Apple GABA Shunt Gene Family

**Figure 1:**
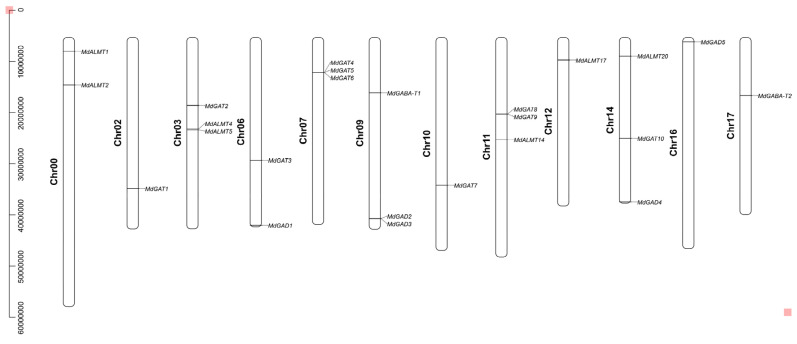
Chromosome distribution of the GABA pathway genes in apple.

The 24 GABA pathway genes were widely distributed in 11 chromosomes of the apple genome (Figure 1). Of these genes, five *MdGAD* genes were located on four chromosomes (6, 9, 14 and 16); two *GABA-T* genes were located on two chromosomes (9 and 17); 10 *MdGAT* genes were located on seven chromosomes (2, 3, 6, 7, 10, 11 and 14); and seven *MdALMT* genes were located on four chromosomes (0, 3, 11, 12 and 14). More GABA pathway genes were detected on chromosome 3, 7, 9, 11 and 14; two genes each on chromosomes 0 and 6, and gene on each of the remaining chromosomes.

**Figure 2:**
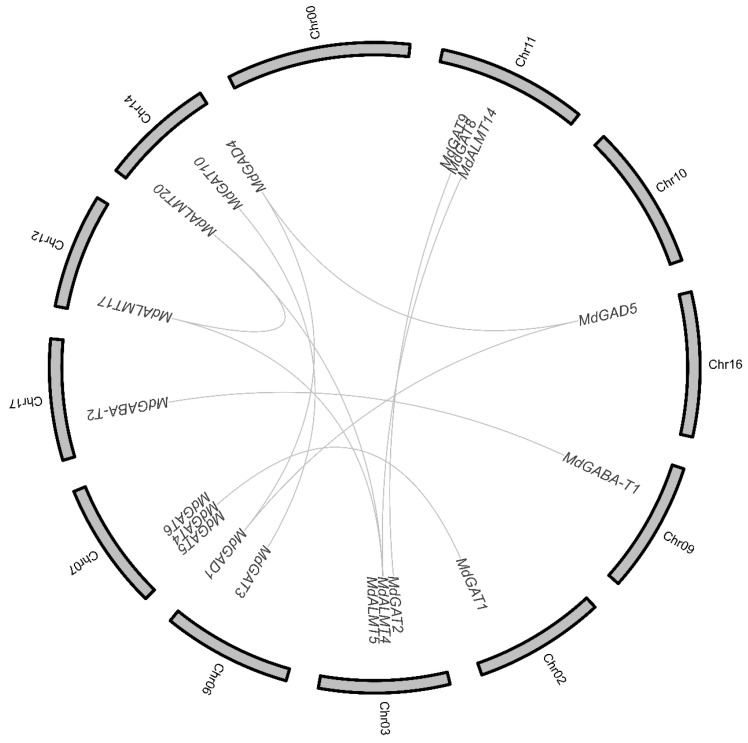
Collinearity analysis of GABA pathway genes in apple.

It has been confirmed that whole-genome duplication and segmental duplication occurred during the process of apple domestication [30,38] (Jung et al., 2019; Velasco et al., 2010). Subsequently, whole-genome duplications and segmental duplications (WGD/segmental duplication) of the GABA pathway genes were analyzed (Figure 2). A total of 19 (79%) GABA pathway genes, including three *MdGAD* gene, two *MdGABA-T* gene, nine *MdGAT* gene and five *MdALMT* gene in apple exhibited WGD/segmental duplication. These observations suggest that WGD/segmental duplication played an important role in the expansion of the apple GABA pathway genes family, as this process allowed the retention of numerous duplicated genes in the genome.

Ten pairs of paralogous GABA pathway genes were identified and distributed on different chromosomes in apple, whereas no tandem duplication events were observed, suggesting that segmental duplications were the main causes for the amplification of GABA pathway gene family.

In addition, there are three genes involved in two segmental duplication events (*MdALMT4*/*MdALMT17*/*MdALMT20*).

### 3.3. Evolutionary Relationships of GABA Shunt Genes between Apple and Arabidopsis

**Figure 3:**
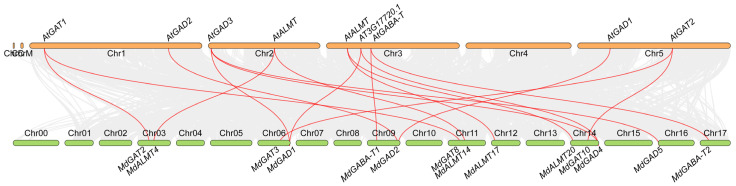
The comparative synteny map of GABA shunt genes between apple and Arabidopsis genomes.

To further explore the origin and evolutionary process of apple GABA shunt genes, we performed the comparative synteny map between apple and Arabidopsis genomes. According to comparative genomics, we can determine the origin and diversification of apple GABA shunt genes based on their Arabidopsis homologs. Large-scale syntenies contained 14 GABA shunt genes in apple and 9 GABA shunt genes in Arabidopsis (Figure 3 and Table S2). Among these syntenies, only one was unambiguous: *MdGAD5*-*AT2G02000*. More challenging for syntenic interpretation were cases where apple segmental duplications corresponded to a single Arabidopsis gene or where a single apple gene corresponded to multiple Arabidopsis genes. The first situation included *MdGABA-T1/MdGABA-T2*-*AT3G22200*, *MdGAT2/MdGAT8*-*AT1G08230*, *MdGAT3/MdGAT10*-*AT5G41800*, *MdALMT4/MdALMT14*-*AT2G27240*, *MdALMT17/MdALMT20*-*AT3G11680*; whereas the second included *MdGAD2*-*AT1G65960/AT5G17330*. Finally, a third case was identified where two duplicated apple genes corresponded to two Arabidopsis genes: *MdGAD1/MdGAD4*-*AT2G02000/AT3G17720*.

### 3.4. Gene Structure Analysis and Conserved Motif Identification in Apple GABA Pathway Genes

**Figure 4:**
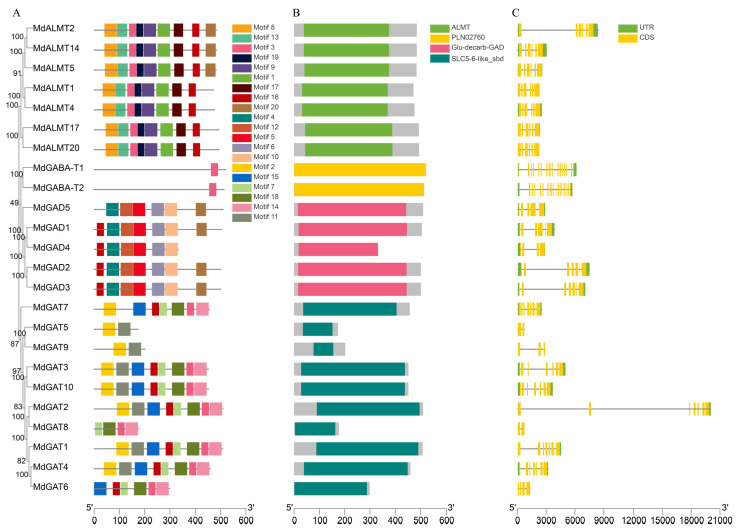
Phylogenetic relationships, motif composition, conserved domains, and gene structures of GABA pathway genes. (**A**) Conserved motifs analysis of MdGAD, MdGABA-T, MdGAT and MdAMLT proteins using MEME tools. Conserved motifs are showed in different colored boxes. (**B**) Functional domain analysis of MdGAD, MdGABA-T, MdGAT and MdAMLT proteins. (**C**) The intron/exon organization in GABA pathway genes is represented; yellow boxes depicting exons separated by introns with thin lines. Green boxes indicated UTRs.

To further study the conserved motif and structural features of the GABA pathway protein in apple, the gene structures, and conserved motifs of *MdGAD*, *MdGABA-T*, *MdGAT* and *MdALMT* were investigated. A total of 20 motif were identified in apple GABA pathway protein. Furthermore, MdGAD proteins have seven motifs, five of them were conserved; only one conserved motif displayed on MdGABA-T protein; eight motifs were found in MdGAT proteins, but none of them were conserved; nine motifs displayed on MdALMT and eight of them were conserved (Figure 4A). MdGAD, MdGABA-T, MdGAT and MdAMLT proteins demonstrated similar conserved motif composition, which was in agreement with the gene structure analysis. Specifically, the results showed that the highly conserved Glu-decarb-GAD domain existed in five MdGAD proteins; PLN02760 domain existed in two MdGABA-T proteins; SLC5-6-like-sbd domain was existed in two MdGAT proteins and ALMT domain existed in seven MdALMT proteins (Figure 4B). Almost all GABA pathway genes exhibited highly conserved exon-intron organization. Some special cases were observed; among five *GAD* gene family, *GAD2* and *GAD3* gene have a long intron, respectively. *MdGAT2* gene, which was longest among seven *MdGAT* family genes, has two long introns. In addition, the construction of *MdALMT2* gene, which has one long intron, was different from the other six *MdALMT* gene (Figure 4C).

### 3.5. Expression Profiles of GABA Pathway Genes in Different Tissues Development, Biological and Abiotic Stress

**Figure 5:**
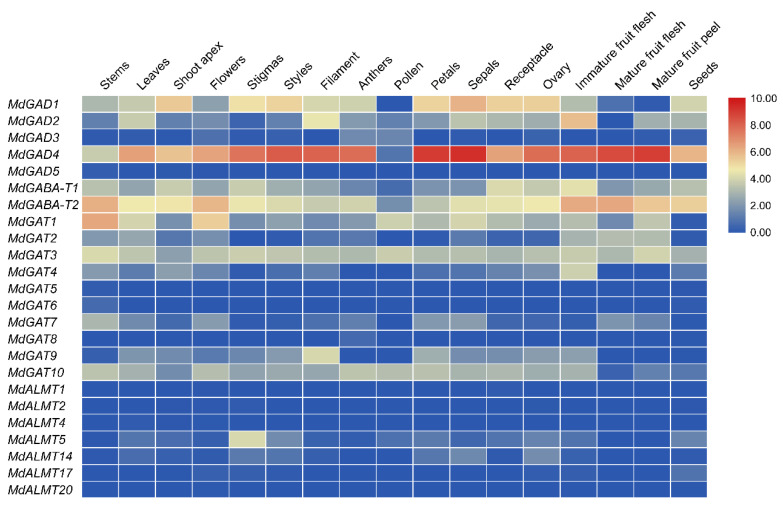
Heat map of GABA signal pathway genes in the stem, leaves, flower organ, fruit and seed tissues. Relative expression values with color blue (low) to red (high) are displayed at the bottom.

The tissue-specific expression of 24 GABA signal pathway genes was determined to compare their expression levels with stem, leaves, flower organ, fruit and seed tissues of apple through website information (http://bioinformatics.cau.edu.cn/AppleMDO, 15 November 2020) [39]. The results showed that (Figure 5 and Table S3), among 5 *GAD* genes, *MdGAD4* was obviously expressed in leaves, shoot apex, flower organs, fruit, and seeds, especially, in petals, sepals and mature fruit peel. The expression level of *MdGAD1* was higher in the shoot apex and flower organs and the expression level of *MdGAD2* was obviously increased only in immature fruits, while the gene expression level of *MdGAD3* and *MdGAD5* were not obviously expressed in different organ tissues. The *MdGABA-T2* transcript level was higher in stem, flower, and fruit, but *MdGABA-T2* transcript level was low in all tissues except for a slight increase in the receptacle and immature fruit. The expression of *MdGAT* and *MdALMT* family genes remained low level in all tissues except *MdGAT1* and *MdGAT1*, which expression was obviously increased in the stem, flower organ, fruit, and seed tissues. These important indices concerning similarities and differences among the 17 tissue expressions suggested that *MdGAD* family genes may be involved in floral organ and fruit development.

**Figure 6:**
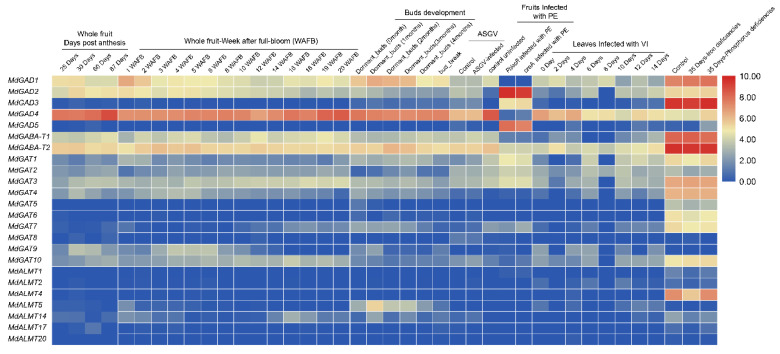
The expression of 24 GABA signal pathway gene involving growth and developmental process of apple were investigated use the website. Relative expression values with color blue (low) to red (high) are displayed at the bottom.

In addition, the expression of 24 GABA signal pathway gene involving the growth and developmental process of apple were investigated use the website (http://bioinformatics.cau.edu.cn/AppleMDO, 15 November 2020) [39]. Meanwhile, the GABA signal pathway gene, which responded to several biological and abiotic stresses, was investigated in a previous study [40]. The result (Figure 6 and Table S4) showed that most of GABA signal pathway gene has no significant expression during the fruit development process, except for *MdGAD1* and *MdGAD4*. Specifically, a slightly higher expression of *MdGAD1* in the first and second week after full bloom was observed, which subsequently declined from the third to the twentieth week; the expression of *MdGAD4* showed a slight increase at 87 days post-anthesis and twentieth week after full bloom. During buds’ development and growth process, among 24 GABA signal pathway genes, the *MdGAD1*, *MdGABA-T2* and *MdAMLT5* showed a similar expression pattern with an increase and then, subsequently, a decline from initiation of dormant buds to bud break. Based on the results above, we further acknowledge that GABA maybe play an important role during fruit and buds’ development.

The treatments of Apple Stem Grooving Virus (ASGV) infected iron and phosphorus deficiencies are commonly biological and abiotic stresses in plant development [40]. The expression of 24 GABA signal pathway genes has no significant difference in these treatments. Additionally, there is no obvious variation pattern of these genes after the treatment of leaves infected with *V*. *inaequalis* (VI). Unexpectedly, the expression of GABA pathway genes has significant variations in the treatment of fruit infected with *P*. *expansum* (PE). To be more specific, the expression of *MdGAD2*, *MdGAD3* and *MdGAD5* were significantly up regulated after fruit infected with PE. On the contrary, a significant down-regulation in the gene expression of *MdGAD1* and *MdGAD4* after fruit was infected with PE was observed. The up-regulation expression of *MdGABA-T1* and the down-regulation expression of *MdGABA-T2* after fruit infected with PE treatment was also observed. In view of the above results, we indicated that the GABA signal pathway gene plays a core role in floral organ, fruit and buds development, especially of the GAD and GABA-T gene family.

### 3.6. Cis-Acting Elements in the Promoter of the GABA Pathway Genes

**Figure 7:**
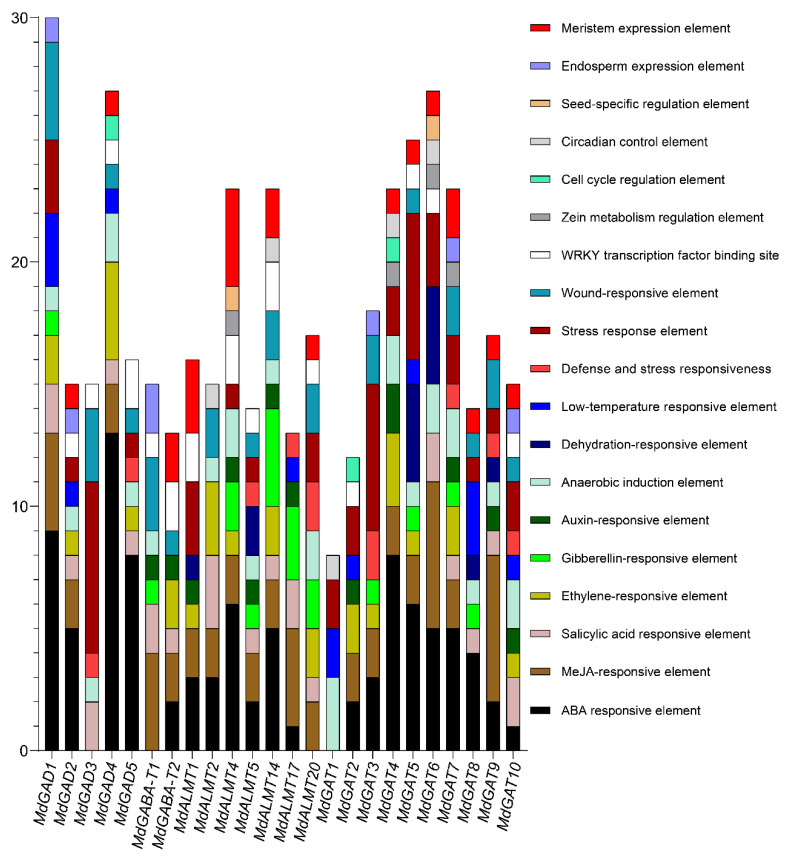
Illustration of cis-regulatory units in all promoters of the GABA signal pathway gene in apple. From the translation start site, the upstream region 2000 bp of each GABA signal pathway genes promoter.

For further insight into the genes function and regulation mechanism of GABA signal pathway, including *MdGADs*, *MdGABA-Ts*, *MdGATs* and *MdALMTs*, the cis-acting elements in promoter sequences were analyzed. The GABA signal pathway genes promoters, including 2000 bp of genomic DNA sequence upstream of the translation starts site, were submitted in the PlantCARE database [33]. All cis-acting regulatory components for each single promoter sequence were identified (Figure 7 and Table S5).

In hormone-related cis-acting elements, the ABA responsive element (ABRE), the MeJA-responsive element (CGTCA-motif and TGACG-motif), the salicylic acid (SARE and TCA-element), and the ethylene-responsive element (ERF) were identified in the promoter region of the most of GABA pathway genes. Gibberellin-responsive element (P-box, GARE-motif, and TATC-box) and auxin-responsive element (AuxRR-core and TGA-element) were observed in the promoter of *MdGABA-T*, *MdGAT* and *MdALMT* genes, but none of *MdGAD* genes promoter was observed (Figure 7 and Table S5). Lots of hormone-responsive elements were observed in the promoter region of GABA pathway genes, revealing a close relationship with GABA and hormones and also reconfirming that the GABA signal pathway could play important functions in the regulation of plant growth and development.

In stress-related cis-acting elements, anaerobic induction (ARE), defense and stress responsiveness (MBS), stress response (HSE) and low-temperature (LTR) responsiveness elements were also detected in the promoters of *MdGAD*, *MdGABA-T*, *MdGAT* and *MdALMT* genes. Meristem expression (CAT-box, CCGTCCbox and dOCT) and endosperm expression (GCN4_motif and Skn-1_motif) were found in the promoter of GABA signal pathway genes. Unexpectedly, the promoter of GABA signal pathway gene, which includes *MdGADs*, *MdGABA-Ts*, *MdGATs* and *MdAMLTs*, was rich in the WRKY transcription factor binding site (Figure 7 and Table S5).

## 4. Discussion

GABA, as a signaling molecule, plays a crucial role in plant growth and development, biological and abiotic stresses, pH regulation and maintaining C/N balance, etc. [3,5,6,41]. There have four types of GABA signal pathway genes, namely GAD, GABA-T, GAT and ALMT that are involved in the synthesis, metabolism, transport, and receptor of GABA, respectively. However, there are no reports on the characterization of apple GABA signal pathway genes despite the availability of the apple genome sequence. In this study, we performed a genome wide analysis and expression profiling of the GABA pathway gene family in the apple genome. These results can serve as a guide for future studies on the understanding and functional characterization of these gene families.

GAD, an essential rate-limiting enzyme, catalyzes glutamate converting to GABA and is widely distributed in organisms [7,42]. In a previous study, the gene code GAD enzymes have been successfully identified by RACE method in many species, such as Arabidopsis [43], maize [44], and also in some woody plants, such as apple [45], citrus [42], tea [46] and *Caragana intermedia* [47]. However, a comprehensive analysis of the GAD gene by bioinformatics was not well understood. In addition, several studies have shown that different GAD gene family members have different expression patterns. For example, AtGAD1 is mainly expressed in Arabidopsis roots [43], while ZmGAD1 is expressed in the leaves, stems, and roots of maize [44]. In rice, OsGAD1 is mainly expressed in seeds, but OsGAD2 is mainly expressed in roots [48]. In citrus, CsGAD1 is predominantly expressed in flowers, but CsGAD2 is predominantly expressed in fruit [42]. These studies also indicated that GADs might play important roles in plant development and stress responses. In this current study, five GAD genes were identified in the apple genome, among the five GAD genes, *MdGAD4* was obviously expressed in leaves, shoot apex, flower organs, fruit, and seeds, especially, in petals, sepals and mature fruit peel. Additionally, the expression level of *MdGAD1* was higher in the shoot apex and flower organs and the expression level of *MdGAD2* was obviously increased only in immature fruits. For the developmental phase, the expression of *MdGAD4* was obviously higher in fruit and buds’ development than other *MdGAD* family members (Figure 5). The reason for this might be that the promoters of the *MdGAD1*, *MdGAD2* or *MdGAD4* contain more phytohormone, metabolism and development-related cis-elements than those of *MdGAD3* or *MdGAD5* (Figure 7). In addition, the five GADs perform differently in response to biological and abiotic stresses and the expression of *MdGAD2*, *MdGAD3* and *MdGAD5* were obviously up regulated after fruit infected with PE. On the contrary, a significant down-regulation in the gene expression of *MdGAD1* and *MdGAD4* after fruit infected with PE was observed (Figure 6).

The efflux malate anions through channels are stimulated by external Al^3+^ ions. This feature of a few proteins determined the name of the entire protein family as Aluminum-activated Malate Transporters (ALMT) [49]. In an interesting study, TaALMT1 acting as a GABA receptor was first found in wheat, resulting in altered root growth and tolerance to Al, acidic, or alkaline pH [15]. This mechanism would help the plant to avoid excessive loss of reduced carbon, which is crucial for plant growth and development under stress [50]. A total of 25 *MdALMT* genes were identified from the apple reference genome of the “Golden Delicious” doubled-haploid tree (GDDH13) [35]. Based on GABA receptor characteristics with 12 amino acids in length, shared between ALMT and the ion channels used to construct the α_1_β_2_γ_2_ GABA_A_ [36,37], seven putative GABA receptors, namely *MdALMT* genes that play core roles in GABA signal transduction, were identified (Table 1 and Figure S1). In previous studies, phylogenetic analyses of ALMT proteins from plants, such as *Arabidopsis thaliana*, *Populus* *trichocarpa*, *Oryza* *sativa*, *Selaginella moellendorffii*, and moss *Physcomitrella* *patens,* subdivided these proteins into five distinct clades [3,51]. Clade 1, 2a, 3, 4, and 5 all have GABA motifs that can be predicted to be GABA sensitive, except clade 2b. The motif region from wheat (TaALMT1), barley (HvALMT1), rice (OsALMT5), and Arabidopsis (AtALMT1), which fall into the evolutionary clade 1, have been shown to be regulated by GABA [15].

## Figures and Tables

**Table 1:** GABA pathway genes identified in this study. (cyto: cytoplasmic; chlo: chloroplast; plas: integral membrane; vacu: vacuole).

Gene Name	Gene Name Abbreviation	Locus	Chromosome	Start Position	End Position	Strand	Length (aa)	pI	MW (kD)	Subcellular Localization Predicted	Potential Functions
Putative glutamate decarboxylase	*MdGAD1*	MD06G1235500	Chr06	36665970	36669767	−	505	5.49	56.50	cyto	GABA Biosynthesis
	*MdGAD2*	MD09G1276300	Chr09	35359458	35366901	−	501	5.62	56.93	cyto	
	*MdGAD3*	MD09G1277000	Chr09	35381876	35388878	−	501	5.71	56.85	cyto	
	*MdGAD4*	MD14G1242700	Chr14	32108140	32110957	−	333	4.95	37.68	cyto	
	*MdGAD5*	MD16G1010800	Chr16	826461	829289	+	510	5.65	56.95	chlo	
Putative GABA transaminase	*MdGABA-T1*	MD09G1139200	Chr09	10792690	10798780	−	521	6.95	57.59	chlo	GABA Catabolism
	*MdGABA-T2*	MD17G1128700	Chr17	11307951	11313609	−	514	6.95	56.69	chlo	
Putative GABA transporters	*MdGAT1*	MD02G1244800	Chr02	29475795	29480278	−	508	9.07	56.22	plas	GABA Transport
	*MdGAT2*	MD03G1133300	Chr03	13280438	13300479	−	510	9.28	56.40	plas	
	*MdGAT3*	MD06G1103100	Chr06	23991060	23995998	+	452	8.91	49.64	cyto	
	*MdGAT4*	MD07G1072100	Chr07	6824513	6827647	+	460	8.75	50.35	plas	
	*MdGAT5*	MD07G1072200	Chr07	6832373	6833039	+	174	4.79	19.42	cyto	
	*MdGAT6*	MD07G1072300	Chr07	6833186	6834458	+	299	9.22	32.55	chlo	
	*MdGAT7*	MD10G1191600	Chr10	28831972	28834442	+	457	8.97	50.79	plas	
	*MdGAT8*	MD11G1155800	Chr11	14931465	14932148	−	177	9.88	19.42	vacu	
	*MdGAT9*	MD11G1156000	Chr11	14949839	14952668	−	202	7.69	22.50	plas	
	*MdGAT10*	MD14G1122000	Chr14	19672859	19676484	+	452	8.97	49.81	plas	
Putative GABA receptors	*MdALMT1*	MD00G1017600	Chr00	2696703	2698931	−	472	8.21	51.81	plas	GABA Receptors
	*MdALMT2*	MD00G1049200	Chr00	9253069	9261444	+	485	9.28	53.69	plas	
	*MdALMT4*	MD03G1155200	Chr03	17835181	17837669	+	476	8.73	52.20	plas	
	*MdALMT5*	MD03G1155400	Chr03	18018465	18020999	+	484	8.83	53.34	plas	
	*MdALMT14*	MD11G1173000	Chr11	19922876	19925870	+	485	9.39	53.75	plas	
	*MdALMT17*	MD12G1040500	Chr12	4405915	4408221	−	493	8.30	53.72	plas	
	*MdALMT20*	MD14G1039500	Chr14	3629641	3631853	−	494	8.84	53.85	plas	

## Data Availability

Data is contained within the article or supplementary material, further inquiries can be directed to the corresponding author.

## Supplementary Materials

The following are available online at https://www.mdpi.com/article/10.3390/genes12121973/s1, Figure S1: Analysis of gene and protein sequences structure and conserved motif of *MdALMTs*, Table S1: Promoter, CDS, and Protein sequences of GABA signaling pathway genes, Table S2: Synteny analysis of apple and Arabidopsis GABA signaling pathway genes, Table S3: FPKM of *M. domestica* GABA signaling pathway genes in the stem, leaves, flower organ, fruit and seed tissues, Table S4: FPKM of *M. domestica* GABA signaling pathway genes in different growth and developmental process, Table S5: Cis-elements in promoter region of *M. domestica* GABA signaling pathway genes.
