# Supplementary figures and images for: Comprehensive Genome-Wide Identification and Transcript Profiling of GABA Pathway Gene Family in Apple (Malus domestica)
Source: Genes (Basel). 2021 Dec 12;12(12):1973. doi: 10.3390/genes12121973 (PMC8700813; doi:10.3390/genes12121973)

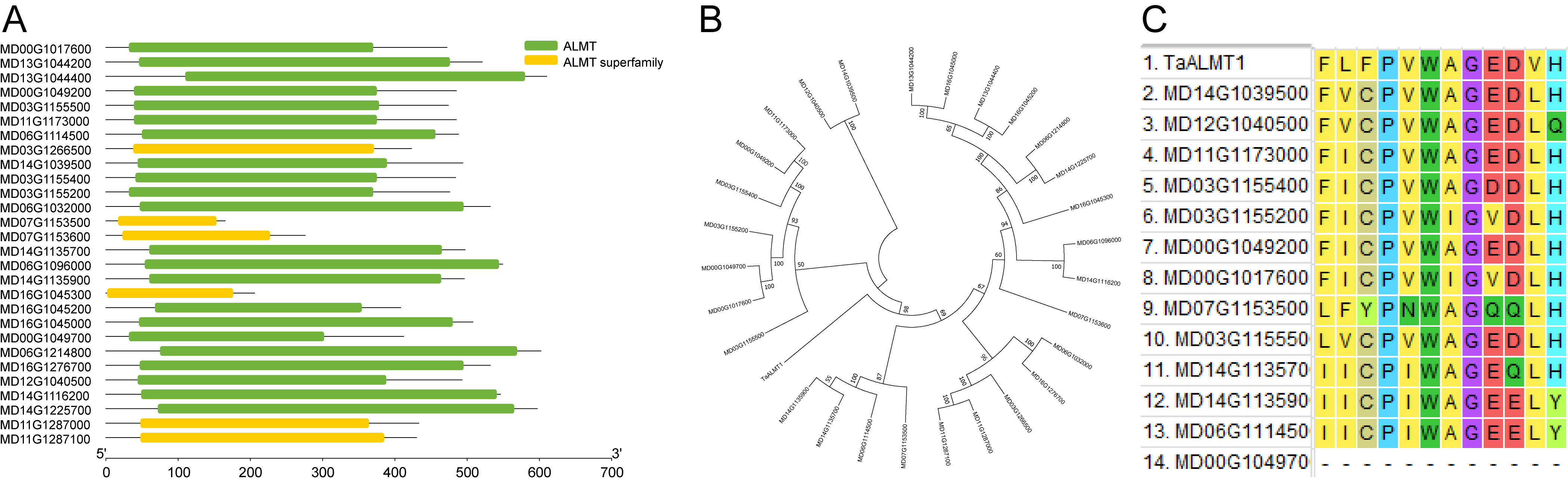

Supplement: Supplementary file 1 [file genes-12-01973-s001.zip › supplementary/Supplemental Figure 1-MdALMT identify.tif]
